# Editorial: Preparation of functional materials and utilization of renewable resources in green solvents

**DOI:** 10.3389/fchem.2022.1085405

**Published:** 2022-11-14

**Authors:** Honglei Fan, Jinliang Song, Hongliang Liu, Zhenyu Sun, Zongyu Wang

**Affiliations:** ^1^ College of Chemistry and Chemical Engineering, Yantai University, Yantai, China; ^2^ School of Chemical Engineering and Light Industry, Guangdong University of Technology, Guangzhou, China; ^3^ College of Chemical Engineering, Beijing University of Chemical Technology, Beijing, China; ^4^ Oak Ridge National Laboratory, Oak Ridge, TN, United States

**Keywords:** green solvent, renewable resource, catalysis, functional materials, modification

The increasing consumption of toxic and non-renewable materials compels researchers to replace them with less dangerous, bio-based, and renewable materials. Moreover, legislative changes have implemented restrictions on the use of commonly employed dipolar aprotic solvents (e.g., dimethylformamide and *N*-methyl-2-pyrrolidinone) and ethers (e.g., 1,4-dioxane) (Jordan et al., 2022). Therefore, the employment of green solvents is an effective way to achieve sustainable development. To realize this, green solvents are regularly used nowadays and include water, alcohols, ionic liquids, deep eutectic solvents, and supercritical fluids. Green solvents are excellent solvents for a variety of chemical processes, for example, the preparation of functional materials, catalysis, and organic synthesis. Additionally, purification and extraction require large excesses of solvents and large amounts of energy. The utilization of green solvents may provide an economic and environmental way to realize environmental sustainability. This Research Topic highlights new research on the employment of green solvents in the preparation of functional materials and improvements in the catalytic efficiencies of important chemical reactions.

Green, task-specific solvents are advantageous in the fabrication of functional materials exhibiting controllable properties. Several studies in this Research Topic are related with this attractive aspect. Polymeric membrane fabrication should satisfy the principles of green chemistry since, generally, a wide range of toxic solvents are used for polymer dissolution and modification (Mehrabani et al., 2022). Hou et al. reviewed the modification of poly (3,4-ethylenedioxythiophene) (PEDOT) in green solvents. For example, alcoholic solvents could replace organic solvents like *N*-methyl-2-pyrrolidone and pyridine to afford PEDOT almost consistent conductivities. High boiling point polar solvents generally increased the reaction time for forming longer polymer chains, allowing higher conductivities to be achieved. Biopolymers are excellent candidates for the fabrication of sustainable materials owing to their excellent availabilities, renewabilities, biodegradabilities, and biocompatibilities. Chemical modification is an appealing way to broaden the utilization of biopolymers (Ge et al., 2022). Li et al. designed adhesives for underwater use. As opposed to bonding in air, underwater bonding is quite challenging. Smart adhesives were explored and exhibited great potential in removing interfacial water and enhancing cohesion by using special functional groups. You et al. replaced sulfuric acid with a combination of carbon dioxide and water for the *in-situ* formation of acidic solutions, thereby rendering the fabrication process for porous silica gel green.

Solvents have a significant impact on reaction efficiencies (i.e., the activity and selectivity), which can be determined in numerous ways, such as the solubilities and stabilities of transient states (Hessel et al., 2022). In relation to this interesting aspect, Jia et al. prepared Cu/SiO_2_ in different aqueous solutions. The as-prepared catalysts were synthesized using a hydrothermal method and showed high reaction selectivities due to high Cu dispersions, small particle sizes, and high proportions of metallic Cu^0^. Water, the most abundant and important solvent on earth, is cheap, green, readily available, and can be used to produce hydrogen or oxygen through electrolysis (Yu et al., 2022). Hui et al. reviewed the synthesis of ammonia by nitrogen photo-reduction over tungsten and the related metal semiconductors. For this transformation, water played dual roles as a hydrogen resource and a reaction medium, and the entire process was also promoted by water oxidation. Wang et al. developed a green process for the efficient hydrodeoxygenation of lignin-derived phenolic compounds in water. Using bio-alcohols as a hydrogen source can not only avoid risks related to high-pressure hydrogen, but can also improve the reaction efficiencies. Hao et al. enhanced the hydrogenation efficiency of furfural over a carboxymethyl cellulose zirconium-based catalyst by using isopropanol as the hydrogen source and the reaction solvent. Wang et al. adopted a similar strategy to promote the transfer-hydrogenation of biomass-derived ethyl levulinate. In addition, the transformation of small molecules like CH_4_ could be realized by thermal, optical, and electrical methods. A numerical simulation of the transformation of CH_4_ benefited the design of catalysts as well as optimization of the reaction process. Zhao et al. revealed a different mechanism for converting methane, exploiting the catalytic roles played by transition metal ions.

The separation of mixtures of miscible liquids is often a tedious and high energy-consumption process. Thus, it is still a major challenge to find an efficient and high-flux strategy. Wei et al. reported a superwetting membrane system to enrich bio-ethanol from water using a novel high-flux method. Excellent performance in obtaining concentrated ethanol was realized by synergistically regulating both the surface energy of the solid porous membrane, and the hydration between an additive inorganic potassium salt and water.

In this Research Topic, we have described recent developments and innovative technologies in the fabrication of functional materials. We have also discussed the performances of important catalytic transformations in green solvents, with the purpose of promoting the utilization of green solvents in related areas.
